# Can the Follicle-Crown Ratio of the Impacted Third Molars be a Reliable Indicator of Pathologic Problem?

**Published:** 2014-12

**Authors:** Sina Haghanifar, Ehsan Moudi, Maryam Seyedmajidi, Mohammad Mehdizadeh, Kamran Nosrati, Naghi Abbaszadeh, Ali Bijani, Hakimeh Ghorbani

**Affiliations:** a Dept. of Oral & Maxillofacial Radiology and Dental Material Research Center, School of Dentistry, Babol University of Medical Sciences, Babol, Iran.; b Dept. of Oral & Maxillofacial Pathology and Dental Material Research Center, School of Dentistry, Babol University of Medical Sciences, Babol, Iran.; c Dept. of Oral & Maxillofacial Surgery and Dental Material Research Center, School of Dentistry, Babol University of Medical Sciences, Babol, Iran.; d Oral & Maxillofacial Radiologist, Babol, Iran.; e General Practitioner, Non-Communicable Pediatric Diseases Research Center, Babol University of Medical Sciences, Babol, Iran.

**Keywords:** Follicular spaces, Pericoronal pathosis, Radiography, Third molar

## Abstract

**Statement of the Problem:** The presence of impacted third molars in the jaws is a common finding in the routine dental examination of patients. Concerning the odontogenic components of the dental follicle, it can be the origin of different types of odontogenic cysts and tumors.

**Purpose:** The aim of this study was to find feasible radiographic criteria to help differentiate between normal and pathological dental follicles.

**Materials and Method:** 134 asymptomatic impacted third molars were recruited in this study. Then, based on the radiographic measurements, the ratio between the diameter of the dental follicle and the mesiodistal width of the tooth crown was calculated. After surgical removal of impacted third molars, the related dental follicles were evaluated histopathologically. Statistical analyses were performed by adopting chi-square test, t-test, receiver oprating characteristic (ROC) curve, and logistic regression using SPSS-19 software.

**Results:** The mean ratio of the dental follicle’s diameter to the mesiodistal width, in the normal and cystic follicle group was 1.18 ± 0.07 and 1.18 ± 0.08, respectively. There was no statistically significant difference between this ratio and the histopathological evaluation. Based on the logistic regression analysis, only the age >20 years and inflammation had predictive value in identifying cystic changes in dental follicle.

**Conclusion:** According to the findings of the current study, the ratio of dental follicle diameter to the mesiodistal width of the teeth cannot not be employed as a diagnostic index to differentiate between normal and pathological dental follicle.

## Introduction

Impacted teeth are a common finding in the routine dental examinations. Some etiologic factors are concerned for the tooth impaction such as insufficient space in the dental arch, malposition, and absence of eruption force.[1] Inflammation and infection, non-restorable caries, cysts and tumors, as well as destruction of adjacent teeth are the relevant indications stated for the extraction of impacted teeth.[2]

Dental follicle is one of the components of tooth germ, which encloses the crown of the impacted tooth and has an imperative role in growth and eruption of teeth.[3-4] In the radiographs, dental follicle demonstrates as pericoronal radiolucency hallow with a 2-2.5 mm width. Radiolucency with more than 2-3 mm width could be considered as an indicative of pathologic change in the dental follicle.[5-6] Radiographic examination is used to identify the presence or absence of any pathosis associated with impacted teeth. Numerous studies have revealed that the prevalence of pathological changes in asymptomatic dental follicles with normal radiographic appearance was much higher than what was expected.[7-9]

Concerning the odontogenic components of the dental follicle, this part of tooth germ can be the origin of different types of odontogenic cysts and tumors;[10-11] among which the dentigerous cysts, keratocystic odontogenic tumor, and ameloblastoma are the most common.[7, 12-13]

Immuno-histochemical studies showed that the dental follicle cells have a great potential for growth and proliferation.[14-16] The dental follicle width has been considered as a measurement for determining the presence or absence of pathology, a method that does not seem to be very reliable. Concerning the different magnification rate in the analog and digital panoramic and periapical views, a definite number cannot be determined. Substantial limitations and different magnification in various radiographic systems of panoramic radiography is completely apparent. The main purpose of the current research was to find a reliable radiographic method to predict cystic changes in dental follicles, being feasible in different radiographic techniques. To overcome these shortcomings, a numerical ratio was proposed in this study. Employing this ratio, if applicable, would help clinicians differentiate normal from pathological dental follicles.

## Materials and Method

In this cross-sectional study, the patients were selected from the referees to the maxillofacial surgery clinics of Babol, Iran. The participants should have been the diagnosed and confirmed candidates for the impacted tooth surgery. Moreover, the participants should have performed related radiological examination, having a panoramic and a periapical (analog and digital) radiographic image with good quality.

When the radiographs showed tooth rotations in which the mesial or distal border of the dental follicle was difficult to be detected, the participants were excluded from the study.

**Figure 1:**
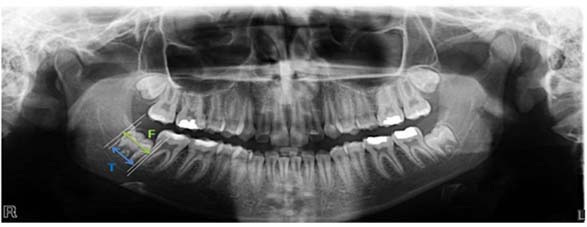
Radiographic measurement for the F/T ratio: follicle diameter (F) mesiodistal width of impacted tooth crown (T).

After recording the patients’ demographic information, a transparent paper was placed on the radiographs of each patient and fixed. Mesial and distal heights of contours of impacted teeth were determined. A line was drawn between these two points and then two parallel lines, tangent to height of contour of the impacted teeth and perpendicular to the mentioned line was plotted (Figure 1).

The distance between the lines was measured by employing a digital caliper (Guanglu; China) with an accuracy of 0.01 mm. The ratio between the dental follicle diameter and mesiodistal width of the tooth crown has been calculated (Ratio= F/T) (Figure 1). The measurements were performed by two expert radiologists and the average values were recorded.

Then, under local anesthesia, the dental follicle and the associated tooth was surgically removed and then fixed in 10% formalin immediately. 

The fixed tissue samples were embedded in the paraffin and cut into 5-micrometer sections by a rotary microtome, and stained with Hematoxylin and Eosin (H&E) solution. An expert oral pathologist, blinded to the clinical and radiological features, performed the histopathological evaluation, observing the stained specimens by using a light microscope (Olympus BX41; Japan). The diagnoses of follicles, cysts or other pathosis were accomplished and recorded. Data were statistically scrutinized by adopting chi-square, logistic regression-test and receiver oprating characteristic (ROC) curve , using the SPSS-19 software. 

## Results

From the 134 dental follicles, obtained by surgery, 37 samples were taken from men (27.6%) and 97 samples were taken from women (72.4%). The mean age of patients was 22.5±5.5 with an age range of 15-43 years. The mean age for men and women was 22.08±5.03 and 22.75±5.74, respectively. Eighty-eight (65.7%) third molars were extracted from mandible and 46(34.3%) from maxilla, 55 samples were taken from the right side of the jaws and 79 from the left. From 134 specimens that were examined histopathologically, 44 cases (32.8%) had undergone cystic changes; all of them were dentigerous cyst. Chronic inflammation was observed in 26(19.4%) samples in dental follicles and dentigerous cysts. 

The average ratio of the dental follicle diameter to the mesiodistal width of the teeth was 1.18±0.07 with a minimum of 1.03 and a maximum of 1.45, respectively. The average ratio of the dental follicle diameter to the mesiodistal width in normal and cystic samples is summarized in Table 1. Based on the ROC curve, we could not find the acceptable cut off point and the area under the curve associated with this index was not feasible for the diagnosis of cystic changes (AUC= 0.477 CI 95%: 0.372-0.581).

**Table 1:** Evaluation criteria for radiographs and histopathological observations

**Variable**	**Histological assessment**	**Average**	**P value**
Follicle diameter	Follicle	13.28±1.95	0.19
	Cyst	13.77±2.25	
Teeth diameter	Follicle	11.20±1.57	0.15
	Cyst	11.63±1.81	
Follicle diameter/ Teeth diameter ratio	Follicle	1.18±0.07	0.90
	Cyst	1.18±0.08	

Although the average age of men was slightly less than that of women, the incidence of cystic changes was higher in male individuals (40.5% versus 29.9%), although there was no significant relationship between gender and pathological features. Moreover, no significant correlation was found between the presence of cystic changes and the tooth location.

The incidence of cystic changes in the participants older than 20 years was 40.3% and cystic changes were observed in 22.8% patients with age 20 years and below; this difference was statistically significant (*p*= 0.03).

Inflammatory infiltration could be detected in 26 (19.4%) of all samples and there was a significant relationship between inflammation and cystic changes (Table 2).

**Table 2:** Prevalence of inflammatory changes on histopathological specimens

**P value**	**Follicle**	**Cyst**	**Variable**
Presence of inflammation	21(47.7%)	5(5.6%)	< 0001
Absence of inflammation	23(52.3%)	85(94.4%)	

Based on Logistic regression analysis, only in the samples of participants having >20 years old and presence of inflammation the cystic changes in the dental follicles could be predicted (Table 3).

**Table 3:** The role of variables in predicting of pathological changes in dental follicle

**Variable**	**Index**		
	**P value**	**CI 95%**	**Odd’s Ratio**
Gender	2.369	0.904-6.208	0.079
Jaw (maxilla/mandible)	0.895	0.352-2.280	0.817
Age	3.492	1.308-9.320	0.013
Ratio of follicle diameter over tooth	1.909	0.371-9.836	0.439
Difference of follicle diameter and tooth	0.937	0.194-4.538	0.936
Inflammation	22.114	6.630-73.763	< 0.0001

## Discussion

This study showed that in 32.8% of asymptomatic dental follicles, epithelial cystic changes were observed, a finding, which was consistent with the results of previous studies.[5, 10] Mesgarzadeh et al. reported the incidence of pathological changes in the dental follicles to be that 53%.[8]

The study of Kotrashetti et al. reported that the pathological changes were observed in the 58.5% cases of asymptomatic dental follicle.[12] Employing various radiographic criteria for normal radiographic appearance of dental follicles in these studies could be the reason for this difference.

In the current study, all pathological samples were diagnosed as dentigerous cysts. In other studies, other pathological lesions such as ameloblastoma, odontogenic myxoma, Gorlin cyst, and more serious lesions such as malignant fibrosarcoma have also been reported.[8, 12-13, 17] The effects of the lesion on the surrounding structure such as root resorption and tooth displacement can be evaluated in the radiographic examinations, which would be inevitably beneficial in differential diagnosis.

Many reports have indicated the follicular space with a maximum thickness of 2.5 mm could be considered as normal.[5, 7, 10] However, in this study, the geometrical characteristics of each radiographs and a numerical ratio between the diameter of the dental follicle and mesiodistal width has been proposed. Although, the average difference between the normal dental follicle and cystic groups was not statistically significant.

The findings of this study showed that the average diameter of teeth associated with cystic follicular tissue, were slightly more than the normal teeth; therefore, the average diameter of the follicles of these teeth was also slightly more than the normal follicles. Neither of these samples showed statistical significant differences but the probability of cystic epithelial changes might be higher when the dental follicles had wider surface than usual.

Most studies have reported that cystic changes have higher prevalence in the mandible.[8-10] Similar results were obtained in the present study; however, no statistically significant difference was found between maxilla and mandible as reported by the study of Adelsperger et al.[5]

In this study, no association was detected between gender and the presence or absence of normal dental follicle. In previous studies, different predilection for men and women were reported,[5, 9, 13] although the reason for this discrepancy is still unclear.

The relationship between age and the incidence of pericoronal pathosis have been reported in some studies; indicating that the probability of cystic changes in impacted teeth was higher in the patients older than 20 years than the younger age group.[5, 7, 13] This study also found statistically significant relationship between the age >20 years and the possibility of cystic follicles. Regarding these findings, and concerning the complications of surgical removal of impacted teeth in elderly patients, it is recommended that prophylactic surgery should be performed at a younger age.

In present study, there was a statistically significant relationship between cystic changes and the presence of chronic inflammatory infiltrate. Khorasani et al. found a correlation between inflammation and squamous metaplasia.[18] In Damante and Fleury study, 36.1% of dental follicles showed inflammation.[19] The inflammatory infiltration might be present due to the physiological process of tooth eruption and alveolar bone resorption. There are other sources to provoke inflammation such as periodontal problems and pericoronitis of the second molars.[7]

It seems that histological examination is necessary to determine the nature of normal or pathological follicle. More longitudinal study should be performed to use this index with adequate follow-ups to determine how many cases, with typical radiographic features, will undergo cystic changes or not. 

Employing advanced imaging techniques, such as cone beam computed tomography (CBCT) are incredibly recommended for accurate assessment of the size of the lesion and its correlation with histopathologic findings. These advance imaging tools might provide an indication guideline for the surgical removal of the asymptomatic impacted teeth. 

## Conclusion

According to the results of the current study, the ratio of dental follicle diameter to the mesiodistal width of the teeth cannot be feasible as a diagnostic index to differentiate between normal and pathological dental follicle. 
